# Practical Approach to Hormone Replacement Therapy for (Peri) Menopausal Women With Schizophrenia Spectrum Disorders: A Case Series

**DOI:** 10.1093/schbul/sbaf170

**Published:** 2026-03-21

**Authors:** Kirsten E Noot, Bodyl A Brand, Iris M H Hamers, Anne Jetske Boer, Mae H Tol, Iris E C Sommer

**Affiliations:** Department of Psychiatry, University Medical Centre Groningen, Groningen, Groningen 9713 GZ, The Netherlands; Centre for Clinical Neuroscience and Cognition, University Medical Centre Groningen, Groningen, Groningen 9713 GZ, The Netherlands; Department of Psychiatry, University of Oxford, Oxford, Oxfordshire OX3 7JX, United Kingdom; Centre for Clinical Neuroscience and Cognition, University Medical Centre Groningen, Groningen, Groningen 9713 GZ, The Netherlands; Centre for Clinical Neuroscience and Cognition, University Medical Centre Groningen, Groningen, Groningen 9713 GZ, The Netherlands; Centre for Clinical Neuroscience and Cognition, University Medical Centre Groningen, Groningen, Groningen 9713 GZ, The Netherlands; Centre for Clinical Neuroscience and Cognition, University Medical Centre Groningen, Groningen, Groningen 9713 GZ, The Netherlands

**Keywords:** psychotic disorders, menopausal hormone therapy, estrogens, women’s mental health

## Abstract

**Background and Hypothesis:**

Estrogens play a neuroprotective role in schizophrenia spectrum disorders (SSD), and evidence suggests that perimenopausal estrogen decline is associated with symptom exacerbation and increased relapse risk in women with SSD. Hormone replacement therapy (HRT) may mitigate these effects, but clinical and epidemiological data on its use in women with SSD remain limited.

**Study Design:**

We conducted a case series of 5 women (aged 47-53 years) with SSD in perimenopause or postmenopause, who were treated with individualized HRT regimens (transdermal or oral estradiol with oral, vaginal, or intrauterine progestogens) in a psychosis outpatient clinic. The women were monitored for 3 months for psychiatric stability, tolerability, side effects, and menopausal symptoms, using clinical observation and self-report.

**Study Results:**

All 5 women tolerated HRT well, with no serious adverse effects. Three women reported notable improvements in mood, energy, and social functioning. Negative symptoms improved in 4 women, and 1 woman with active positive symptoms experienced partial symptom reduction. Three women reported relief of menopausal symptoms (eg, sleep disturbance, vasomotor symptoms, and joint pain), which was perceived as beneficial to mental stability. All patients continued HRT beyond follow-up.

**Conclusions:**

Hormone replacement therapy appears feasible and acceptable for women with SSD before and after the menopausal transition, with potential benefits for mood and functional improvement and high tolerability. Individualized approaches and safety considerations are essential. This approach may be integrated into routine psychiatric care, though larger studies with standardized outcomes are needed.

## Introduction

Emerging research confirms that estrogens have neuroprotective effects relevant to the pathophysiology of schizophrenia spectrum disorders (SSD).^1-4^ Estradiol, the most potent endogenous estrogen, has various functions in the brain, including acting as an antioxidant, enhancing neuroplasticity, facilitating neuroprotection, and functioning as a key regulator of metabolism.^1-3^ Estrogens also modulate key neurotransmitter systems involved in SSD, including dopaminergic, serotonergic, and glutamatergic pathways.^1^ Estrogens are not a stable factor throughout a woman’s life and may fluctuate significantly during different periods, such as puberty, the menstrual cycle, pregnancy, and menopause. Phases with low estrogens have been associated with both increased vulnerability to psychosis onset and more severe symptoms in women.^1^^,^^5^

A steep and permanent decrease in estrogens occurs during the menopausal transition. Menopause, defined as the cessation of menstruation for 12 consecutive months, is preceded by a progressive decrease in estrogens during the perimenopausal years to less than 10% of premenopausal levels. In Europe, menopause occurs at an average age of 51, with perimenopausal endocrine changes typically starting around the age of 45.^6^^,^^7^ Epidemiological studies have identified a second peak in the incidence of psychosis among women aged 45-49 years, coinciding with perimenopause.^4^^,^^5^ In women with SSD, the decline in estradiol during the menopausal transition has been associated with a worsening of positive and negative symptoms and an increased risk of psychotic relapse.^3^^,^^4^^,^^8^ After reaching menopausal age, women with SSD required more hospitalizations for psychotic relapse compared to younger women and male peers.^3^ These findings have led to growing interest in the menopausal transition as a potential window for targeted hormonal interventions in women with SSD, particularly through the use of hormone replacement therapy (HRT).

Hormone replacement therapy has been prescribed since the 1960s to manage somatic symptoms (eg, hot flashes and night sweats) resulting from hormonal fluctuations during menopause. It can be administered in different formulations and through various administration routes, but typically consists of a form of estradiol, combined with a progestogen in women with an intact uterus. While there were initial concerns regarding the safety of HRT, particularly related to increased risks for breast cancer and thrombosis, it is now generally considered safe for most women, especially when estradiol is applied transdermally.^9-11^

Although there is a lack of clinical trials investigating the potential benefits of HRT in women with SSD of menopausal age, emerging real-world evidence suggests that HRT may help to prevent psychotic relapse in this population. A nationwide cohort study using Finnish registry data found that when women with SSD used HRT between the ages of 40 and 62, their risk of hospitalization for psychosis was 16% lower compared to periods without HRT use. There was no difference in this risk between combined HRT and estrogens-only therapy. The study identified a “window of opportunity,” with significant benefits observed during perimenopause (ages 40-49) and early menopause (ages 50-55), but not in later menopause (beyond age 56).^12^ In addition, a randomized controlled trial found that adjunctive estradiol reduced negative symptoms in women with SSD up to age 46, with the effect driven primarily by those aged 38-46 years.^13^

In this article, we present a case series of 5 peri- and postmenopausal women with SSD who were prescribed HRT as part of their treatment plan. We describe the clinical rationale, prescribing approach, and observed experiences of these women. Through these case descriptions, we provide examples of the practicalities and feasibility of prescribing HRT to women with SSD during or shortly after the menopausal transition.

## Methods

We provide a case series focusing on women aged 47-53 with SSD who were treated in a psychosis outpatient clinic. Patients were initially prescribed HRT for 3 months, while we monitored the effects of HRT use on psychiatric stability, tolerability, side effects, and menopausal symptoms. Symptom changes were described based on clinical observation and patient self-report. As this was not an experimental treatment, all women were prescribed HRT as a part of their routine clinical care and were offered the option of continuing HRT after the follow-up of 3 months. Patients were informed about the goal to describe their treatments and effects and provided written informed consent. Since the data for our study were obtained from routine health care and participation imposed no additional burden on patients, our study did not fall under the scope of medical research for which explicit ethical protocols were required to use data for scientific purposes based on Dutch law. The clinical observations were registered under the UMCG 18064 registration code.

### Treatment

When considering HRT, we used the window of opportunity described by Brand et al. (2024) and only provided this option to women between ages 40 and 55.^12^ Women with a history of breast cancer or other estrogen-sensitive tumors, unexplained vaginal bleeding, active or recent thromboembolic disease, and severe liver dysfunction were not considered for treatment, as these are contraindications for HRT. The prescriptions were based on practical guidelines for menopause care in the Netherlands and the NICE guidelines for menopause.^14^^,^^15^ Transdermal applications of estradiol are preferred over oral formulations for their superior safety profile, particularly their lower thromboembolic risk compared to oral estradiol.^16^^,^^17^ We recommended estradiol in the form of either patches, gels, or sprays, based on patient preferences. For long-term use in women with a uterus, the addition of a progestogen is required to prevent chronic endometrial proliferation. Progestogens can be administered orally, vaginally, or via an intrauterine device (IUD). Not all women respond well to the oral form, as it has been reported to increase anxiety and low mood in some.^18^^,^^19^ Furthermore, evidence indicates that safety profiles are most favorable with methods that minimize systemic exposure.^20^^,^^21^ Therefore, we recommended administration via an IUD, which delivers lower systemic doses of progestogen to the brain.^19^ In the Netherlands, 2 types of levonorgestrel-releasing intrauterine devices (LNG-IUDs) are available: a 52 mg LNG-IUD (Mirena) and a 19.5 mg LNG-IUD (Kyleena).^14^ For perimenopausal women, progestogen delivered via an IUD has the additional advantage of providing effective contraception. However, insertion can be painful and may be unacceptable to some women. In the Netherlands, placement of the LNG-IUD is performed by either a primary care physician or a gynecologist. For women who declined an LNG-IUD, vaginal progestogen capsules (200 mg) or oral combination therapy with estradiol (1 mg) and dydrogesterone (5 mg) [Femoston continu] were considered.

## Results

We provide findings from 5 women who were treated with HRT. Patient demographics, clinical characteristics, and type of HRT are summarized in Table 1. Patients had a median age of 50 years (range 47-53 years). Three women (cases 1, 2, and 4) experienced a first psychotic episode in the years leading up to menopause, at ages 50, 46, and 48, respectively. One woman (case 3) had a first episode at the age of 36 and a second episode with a worsening of psychotic symptoms at age 45. Another (case 5) had her first episode at age 26 and expressed fear of a relapse around menopause following a long period of remission. At the time of first contact, 4 of the 5 women were in stable remission from positive symptoms. One woman (case 3) was experiencing olfactory, gustatory, and auditory hallucinations, along with paranoid thoughts and ideas of reference.

**Table 1:** Patient Demographics, Clinical Characteristics and Hormone Replacement Therapy (*n* = 5)

**Case**	**Age at HRT initiation**	**Diagnosis**	**Psychosis status**	**Antipsychotic medication**	**Menopausal status**	**HRT regimen**
1	53	Unsp. SSD/OPD	Onset age 50. Remission.	Aripiprazole 7.5 mg once daily.	PostM	E-patch 50 mcg/day BIW + LNG-IUD 52 mg
2	47	Schizophreniform disorder	Onset age 46. Remission.	Aripiprazole 15 mg once daily.	PeriM	E-patch 50 mcg/day BIW → 75 μg/day BIW + LNG-IUD 52 mg
3	47	Unsp. SSD/OPD	Onset age 36. Relapse age 45. Active symptoms.	Olanzapine 20 mg once daily.	PeriM	E-patch 50 mcg/day BIW + LNG-IUD 52 mg
4	50	Schizophreniform disorder	Onset age 48. Remission.	Aripiprazole 10 mg once daily.	PeriM	Estradiol 1 mg/dydrogesterone 5 mg orally QD → E-patch 50 mcg/day BIW + vaginal progestogen capsule 200 mg QOD
5	50	Schizoaffective disorder	Onset age 26. Remission.	Zuclopenthixol 16 mg once daily.	PeriM	Estradiol gel 0.06%, 0.75 mg QD + LNG-IUD 19.5 mg

Abbreviations: BIW = twice weekly; E-patch = estradiol patch; LNG-IUD = levonorgestrel intrauterine device; PeriM = perimenopausal; PostM = postmenopausal; QD = once daily; QOD = every other day; Unsp. SSD/OPD = unspecified schizophrenia spectrum or other psychotic disorder

All 5 patients started HRT using individualized administration routes. In 3 women (cases 1, 2, and 3), estradiol was initially delivered via transdermal patches with a starting dose of 50 μg/day, twice weekly, and was combined with an LNG-IUD, which had already been in place in 2 (cases 2 and 3) before initiating HRT. In 1 of these 3 women (case 2), the patch dose was increased to 75 μg per day biweekly after 2 months with limited effect. One woman (case 5) was administered estradiol via gel (0.75 mg daily), due to skin sensitivity to patches, combined with an LNG-IUD. Another woman (case 4) started with oral combination therapy (1 mg estradiol and 5 mg dydrogesterone; Femoston continu), due to aversion to the placement of an IUD and a preference for oral medication. Oral therapy was discontinued after 4 weeks due to a lack of effect, after which she switched to estradiol patches with vaginal progestogen capsules (200 mg every other day).

The IUDs were placed by the general physician and the women (cases 1 and 5) tolerated the placement well. Reported benefits of the IUD included its one-time placement and contraceptive efficacy. All 5 women chose to continue combined HRT beyond the first 3 months. Tolerability of the administration routes was high, with no discontinuations or (serious) side effects reported during the observation period.

In total, 3 of the 5 women (cases 1, 4, and 5) described significant improvements in mood after starting HRT. One (case 1) said she felt more like herself again, while another (case 4) reported feeling in a better mood with a renewed sense of courage. She described having fallen in love after being single for a long time. A third (case 5) noted greater mood stability, and her partner described her as more emotionally balanced. Improvements in negative symptoms were seen in 4 women (cases 1, 2, 3, and 4). Three (cases 1, 2, and 4) reported having more energy, while the fourth (case 3) became more physically active, began attending social events, and expressed interest in returning to volunteer work. One woman with persistent hallucinations (case 3) noted that olfactory hallucinations had disappeared, though auditory hallucinations remained unchanged. There was a reduction in her paranoid ideation and ideas of reference, but delusions did not disappear completely. All women remained stable with no recurrence of psychosis. Three patients (cases 1, 3, and 5) also reported improvements in somatic symptoms of menopause, including reduced night sweats, less joint or muscle pain, and better sleep quality. They noted that this helped better maintain psychiatric stability. These effects were seen within a 3-month period.

## Discussion

This case series highlights the feasibility and acceptability of hormone replacement therapy (HRT) in women with SSD during and after the menopausal transition. Women were interested and open to this option and tolerated combined HRT well. Several women reported improvements in mood, energy, and social functioning, suggesting potential benefits of HRT for mood and negative symptoms. Effects on positive symptoms were difficult to assess, as most women were in remission at baseline. However, in 1 woman with active symptoms, a significant reduction in hallucinations and delusional thoughts was observed. Estradiol, delivered orally or via patch or gel, was well tolerated. A total of 4 of 5 women followed our recommendation for transdermal estradiol. The woman who started with oral hormones (combined estradiol and progestogens) reported no initial improvement, but did experience a marked improvement after receiving the combination of transdermal estradiol and vaginal progestogens. This observation parallels findings in larger samples that combined oral hormones are less beneficial for mental health than the locally applied combination.^22^ As the IUD application of progesterone also provides effective contraception, this may make it an advantageous choice for perimenopausal women. Notably, vaginal or intrauterine application of progestogen was deemed acceptable to all 5 patients. We had concerns about reluctance toward local application of progestogens, as the prevalence of sexual trauma in women with severe mental illness is high.^23^^,^^24^ However, with clear information and shared decision-making, this option was preferred by 4 of 5 women, with the fifth patient opting for vaginal application after first trying systemic hormones.

Safety is an important consideration in the use of HRT. Concerns have been raised about the risks of venous thromboembolism (VTE) and breast cancer. However, these risks vary depending on the formulation and route of administration.^16^ Recent studies indicate that transdermal estradiol, when used for up to 5 years, shows no significant increase in VTE risk, in contrast to oral estradiol.^16^^,^^17^^,^^25^^,^^26^ When evaluating the progestogen component of HRT, norpregnane-derived oral progestogens may increase VTE risk.^10^^,^^26^ In contrast, pregnane derivatives, including synthetic dydrogesterone, have not been associated with increased VTE risk when combined with estradiol.^10^^,^^20^^,^^27^ Furthermore, locally administered progestogens with minimal systemic absorption, such as via an LNG-IUD or vaginal application, have also not been associated with increased VTE risk.^21^^,^^28-30^ With regard to breast cancer, evidence indicates that oral dydrogesterone shows no significant increase in short-term use (<5 years).^31^ The LNG-IUD may be associated with a small increase in breast cancer risk in postmenopausal women. However, any absolute risk with use under 5 years is expected to be very low.^29^^,^^32^^,^^33^ In addition, the LNG-IUD combined with estradiol effectively prevents endometrial hyperplasia for at least 5 years.^29^ Nonetheless, individual risk assessment remains crucial, especially in a population with a higher prevalence of smoking and obesity.^34^^,^^35^

Most current guidelines recommend limiting HRT use to 5 years, due to potential cumulative risks, particularly for breast cancer.^17^^,^^31^ However, with longer experience with transdermal formulas and LNG-IUDs, these recommendations may be adapted to accommodate longer safe treatment in the near future.^10^ Furthermore, HRT also has beneficial effects; when taking all risks and protective effects into account, studies show that HRT, when initiated within 10 years after menopause, is associated with reduced all-cause mortality and reduced risks of osteoporosis, coronary heart disease, diabetes, and dementia.^12^^,^^36^^,^^37^

This case series illustrates that HRT for women with SSD is not a one-size-fits-all intervention. Several adjustments to recommended formulations and dosing were needed to address differences in effects and individual preferences. This aligns with recent evidence showing wide variability in estradiol levels among women receiving standard HRT regimens.^38^ Treatment should be tailored based on clinical response and patient preferences. An individualized approach is essential to optimize safety, tolerability, and therapeutic benefit in this population.

The findings in this study must be interpreted with caution. This study only describes observations from clinical treatment in a small sample, without a comparison group. Symptom changes were described based on clinical observation and patient self-report. In addition, follow-up was relatively short, precluding conclusive evidence of tolerability and acceptability of HRT in the long term. Rather, this study aims to provide guidance on practical application and shows evidence of acceptability and tolerability in women with SSD and subjective benefit, particularly in mood and energy. Future studies should assess efficacy and tolerability of HRT in larger groups of women with SSD, using standardized symptom scales, randomized designs and longer follow-up.

## Conclusion

Our case series illustrates that with adequate counseling, HRT can be implemented within routine psychiatric care for this population. This is important, as women with SSD may be particularly vulnerable to the effects of hormonal decline during menopause, which can exacerbate positive, affective, and negative symptoms. Overall, this case series provides early evidence that HRT with transdermal estradiol and a local progestogen may be a viable and acceptable intervention for women with SSD during the menopausal transition. These findings add to the existing observational evidence supporting the benefits of HRT in women with SSD around the menopausal transition and provide new insights into how HRT may contribute to symptom improvement.
